# Usefulness of a Three‐Dimensional‐Printed Model in the Treatment of Irreducible Atlantoaxial Dislocation with Transoral Atlantoaxial Reduction Plate

**DOI:** 10.1111/os.12961

**Published:** 2021-03-15

**Authors:** Qiang Tu, Hu Chen, Xiang‐yang Ma, Jian‐hua Wang, Kai Zhang, Jian‐zhong Xu, Hong Xia

**Affiliations:** ^1^ Department of Orthopaedics General Hospital of Southern Theatre Command of PLA Guangzhou China; ^2^ Department of Orthopaedics, The First School of Clinical Medicine Southern Medical University Guangzhou China; ^3^ Department of Orthopaedics, Southwest Hospital Third Military Medical University Chongqing China

**Keywords:** Atlantoaxial dislocation, Decompression, Three‐dimensional printing, Transoral

## Abstract

**Objective:**

To evaluate the usefulness of a 3D‐printed model for transoral atlantoaxial reduction plate (TARP) surgery in the treatment of irreducible atlantoaxial dislocation (IAAD).

**Methods:**

A retrospective review was conducted of 23 patients (13 men, 10 women; mean age 58.17 ± 5.27 years) with IAAD who underwent TARP from January 2015 to July 2017. Patients were divided into a 3D group (12 patients) and a non‐3D group (11 patients). A preoperative simulation process was undertaken for the patients in the 3D group, with preselection of the TARP system using a 3D‐printed 1:1 scale model, while only imaging data was used for the non‐3D group. Complications, clinical outcomes (Japanese Orthopaedic Association [JOA] and visual analogue score [VAS]), and image measurements (atlas–dens interval [ADI], cervicomedullary angle [CMA], and clivus‐canal angle [CCA]) were noted preoperatively and at the last follow up.

**Results:**

A total of 23 patients with a follow‐up time of 16.26 ± 4.27 months were included in the present study. The surgery duration, intraoperative blood loss, and fluoroscopy times in the 3D group were found to be shorter than those in non‐3D group, with statistical significance. The surgery duration was 3.29 ± 0.45 h in the 3D group and 4.68 ± 0.90 h in the non‐3D group, and the estimated intraoperative blood loss was 131.67 ± 43.03 mL in the 3D group and 185.45 ± 42.28 mL in the non‐3D group. No patients received blood transfusions. The intraoperative fluoroscopy times were 5.67 ± 0.89 in the 3D group and 7.91 ± 1.45 in the non‐3D group. Preoperatively and at last follow up, JOA and VAS scores and ADI, CCA, and CMA were improved significantly within the two groups. However, no statistical difference was observed between the two groups. However, surgical site infection occurred in 1 patient in the 3D group, who underwent an emergency revision operation of the removal of TARP device and posterior occipitocervical fixation; the patient recovered 2 weeks after the surgery. In 2 patients in the traditional group, a mistake occurred in the placement of screws, with no neurological symptoms related to the misplacement.

**Conclusion:**

Preoperative surgical simulation using a 3D‐printed real‐size model is an intuitive and effective aid for TARP surgery for treating IAAD. The 3D‐printed biomodel precisely replicated patient‐specific anatomy for use in complicated craniovertebral junction surgery. The information was more useful than that available with 3D reconstructed images.

## Graphical abstract

Technique and procedures of TARP. Simulation of atlantoaxial dislocation is shown (A). During the surgery, a TARP plate and two screws were, respectively, installed on C_1_, then a provisional reduction screw was placed in the the vertebrae of axis (B). Next, the TARP specialized retractor was installed between a provisional reduction screw and the complex comprised of the atlas and the plate (C). The atlas was ascending longitudinally and was pushed back by turning the nut on the top of the retractor (D, E). After the reduction of the atlas, two bicortical screws were placed at C_2_ and the reduction screw was pulled out (F, G). Finally, autogenous graft was implanted into the joint cavity (H).

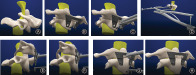

## Introduction

Atlantoaxial dislocation (AAD) is normally characterized by neck pain, limitations in neck movement, and spastic quadriparesis. Surgical intervention is considered a suitable treatment option to restore normal atlantoaxial alignment, relieve spinal cord compression, and provide sufficient stabilization^1^. The atlantoaxial joint is a high‐risk region for surgery owing to its complex anatomy adjacent to important neurovascular structures. For reducible AAD, only posterior decompression and fixation can obtain satisfactory clinical results^2^. However, surgery for irreducible AAD (IAAD) with ventral spinal cord compression remains a technical challenge. Direct anterior decompression of the spinal cord might be performed if traction reduction is not appropriate. At present, the most commonly accepted treatment protocol used to manage IAAD is transoral decompression and soft tissue release combined with posterior fixation^3^. The strategy requires a combined anterior and posterior approach, which increases the risk of surgical‐related injury and perioperative complications. In 2005, Yin *et al*.^4^ first reported on the transoral atlantoaxial reduction plate (TARP) system, which was designed in our institution for the treatment of IAAD. Our previous studies^5^, ^6^, ^7^, ^8^ have confirmed that TARP surgery obtains successfully results in the treatment of IAAD. The TARP surgery makes it possible to perform one‐stage anterior decompression of the ventral spinal cord and internal fixation. It is particularly useful for treating occipitocervical deformities for which posterior stabilization is unsuitable, such as defects of the posterior elements, congenital small pedicles, and “high‐riding” vertebral artery course. However, TARP surgery is technically demanding and is typically performed by experienced surgical teams; surgical mistakes often occur when surgeries are performed by surgeons with insufficient experience. Unacceptable screw placement is a common complication of the TARP procedure.

A good understanding of patients' operative region preoperatively is important for conducting surgery successfully. At present, surgical preplanning for IAAD is based on radiographic examination, including X‐ray, CT, and MRI. However, it is still difficult to distinguish the structure of IAAD because of its complexity. Even though advanced imaging technology can be used to construct realistic 3D digital models, flat 3D images are still two‐dimensional (2D) images created using perspective technology. 3D digital imaging can help to identify the anatomy of the craniocervical junction, but the surgeon still needs to think three‐dimensionally. The added value of a 3D digital model is limited, and diagnostic accuracy is not significantly improved. It is even less helpful for surgical preplanning and accurate screw placement during surgery. Li *et al*. evaluated the accuracy of the placement of screws in TARP surgery in 106 patients and found that combined the ideal and acceptable accuracies for the C_1_ anterior lateral mass screw were 97.6% and those of the C_2_ anterior pedicle screw were 87.0%. The reason for the deviation of the C_2_ screw placement is the variation of the vertebral artery course in the C_2_ lateral mass. Therefore, the surgeons must think about the course of the vertebral artery in a 3D way. How to further improve the surgical efficiency and the accuracy of screw placement in the TARP procedure is a problem that needs to be addressed.

Practice makes perfect, especially for surgeons. Senior surgeons have lots of opportunity to operate on different patients. When a mistake occurs, however, this can lead to great harm to patients, even death. Junior surgeons can identify structures and simulate surgery using cadaver specimens, but this is expensive. In addition, the steep learning curve prevents surgeons from learning the surgery, which may be one of the reasons that TARP surgery is not popular. Reducing the learning curve and finding more practical opportunities has become a challenge in the medical field.

Thanks to advances in 3D‐printing technology, the use of 3D‐printed anatomical models is becoming increasingly widespread in the medical field. A 3D‐printed 1:1 scale model makes it possible for surgeons to obtain a clear understanding of IAAD morphology tridimensionally, which is beneficial for surgical preplanning^9^, ^10^. For this reason, the purpose of this retrospective study is: (i) to investigate the role of 3D‐printed models in understanding the anatomy of the surgical region and in formulating surgical strategies; (ii) to evaluate whether the use of 3D‐printed models can improve the efficiency of the operation, and whether it can help to reduce the operation duration and fluoroscopy time; and (iii) to analyze whether the use of 3D‐printed models can improve the accuracy of screw placement. The present paper explored the feasibility of their clinical application and summarized the application experience for 3D‐printed models using the TARP technique for treating IAAD.

## Materials and Methods

**Fig. 1:**
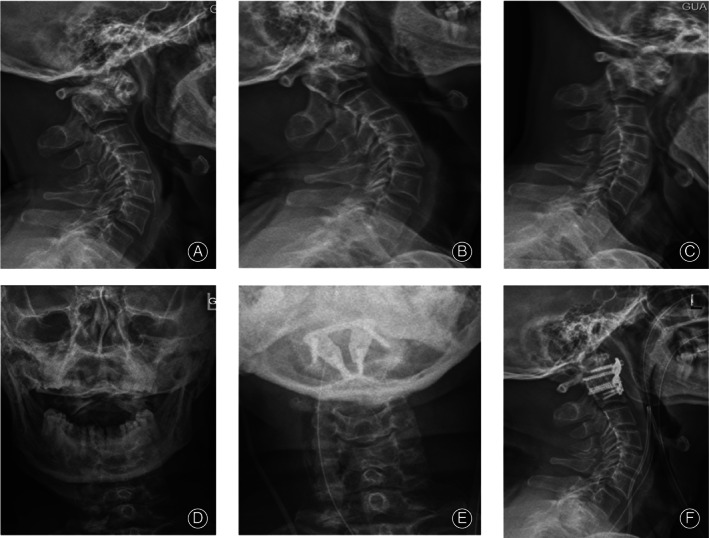
A 62‐year‐old male patient with irreducible atlantoaxial dislocation (IAAD) underwent transoral atlantoaxial reduction plate (TARP) surgery. Preoperative standing lateral (A) and anteroposterior views (D) show that the patient had an atlantoaxial dislocation. In addition, (B) extension and (C) flexion X‐rays show that the atlantoaxial dislocation was irreducible. Postoperative X‐rays show atlantoaxial reduction after TARP surgery (E, F).

**Fig. 2:**
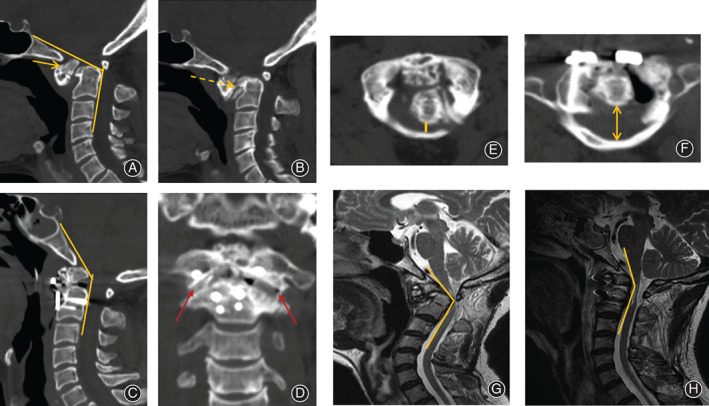
Comparison of CT and MRI preoperatively and postoperatively. (A, C) The clivus‐canal angle (CCA) was significant improved from 107.15° preoperatively to 138.37° postoperatively. Os odontoideum (yellow arrow) was fused with the anterior arch of the atlas. (B) Preoperative CT demonstrated that there was bone connection (yellow dotted line arrow) between the odontoid vertebra and the atlas, and the end of the dens was smooth. (D) Coronal plane CT shows the bone graft (red arrow) in lateral mass joint of bilateral atlantoaxial spine. (E, F) Significant widening of the posterior atlantodental interval from 2.94 mm preoperatively to 15.41 mm postoperatively. (G) Ventral spinal cord compression and myelomalacia could be found in preoperative MRI. (H) Significant improvement of the cervicomedullary angle (CMA) from 118.64° preoperatively to 147.32° postoperatively.

After obtaining the approval of the ethics committee of our institute, patients who had signed an informed consent form were included in the present study. The inclusion criteria were as follows: (i) the atlantoaxial joint had not been reduced after skull traction for 1 week; (ii) absence of oral/retropharyngeal wall or periodontal disease; and (iii) follow up for 12 months or more. Patients who had a history of severe osteoporosis or upper cervical operations were excluded. A total of 23 patients who underwent surgery from January 2015 to July 2017 were finally included. A total of 12 patients (7 male) who underwent surgery assisted by a 3D‐printed model were included; the average age of these patients 58.17 ± 5.27 years. The non‐3D group included 11 patients (6 male) who had undergone the traditional operation; the average age of these patients was 57.45 ± 6.36 years. On admission, all patients received anteroposterior view, lateral view, and cervical dynamic flexion–extension view X‐rays, thin‐slice CT scans, and MR examination (Figs 1 and 2).

### Construction of Three‐Dimensional Digital Models and Surgical Planning

All patients underwent a helical CT scan (64‐slice) from the foramen magnum to the C7 vertebra. The original data (axial images of 0.75 mm thickness, 1.0 mm intervals) acquired from CT scans was saved in DICOM format and imported into specialized software Mimics 14.0 (Materialise, Belgium). All unwanted structures were removed, and a 3D digital model was constructed that included the cervical spine, the craniovertebral junction, and parts of the occiput as well as bilaterally vertebral artery. A 3D digital model can be rotated by any axis and angle. Therefore, the characteristics of abnormalities can be observed omnidirectionally.

**Fig. 3:**
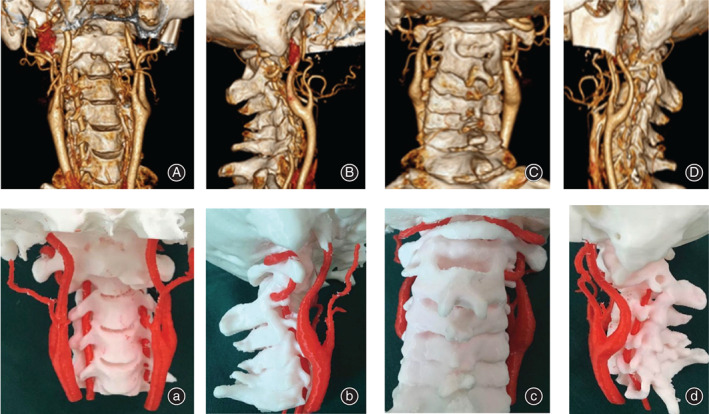
Different view of three‐dimensional (3D) reconstruction imaging (A, B, C, D) and 3D real‐size printed model (a, b, c, d).

**Fig. 4:**
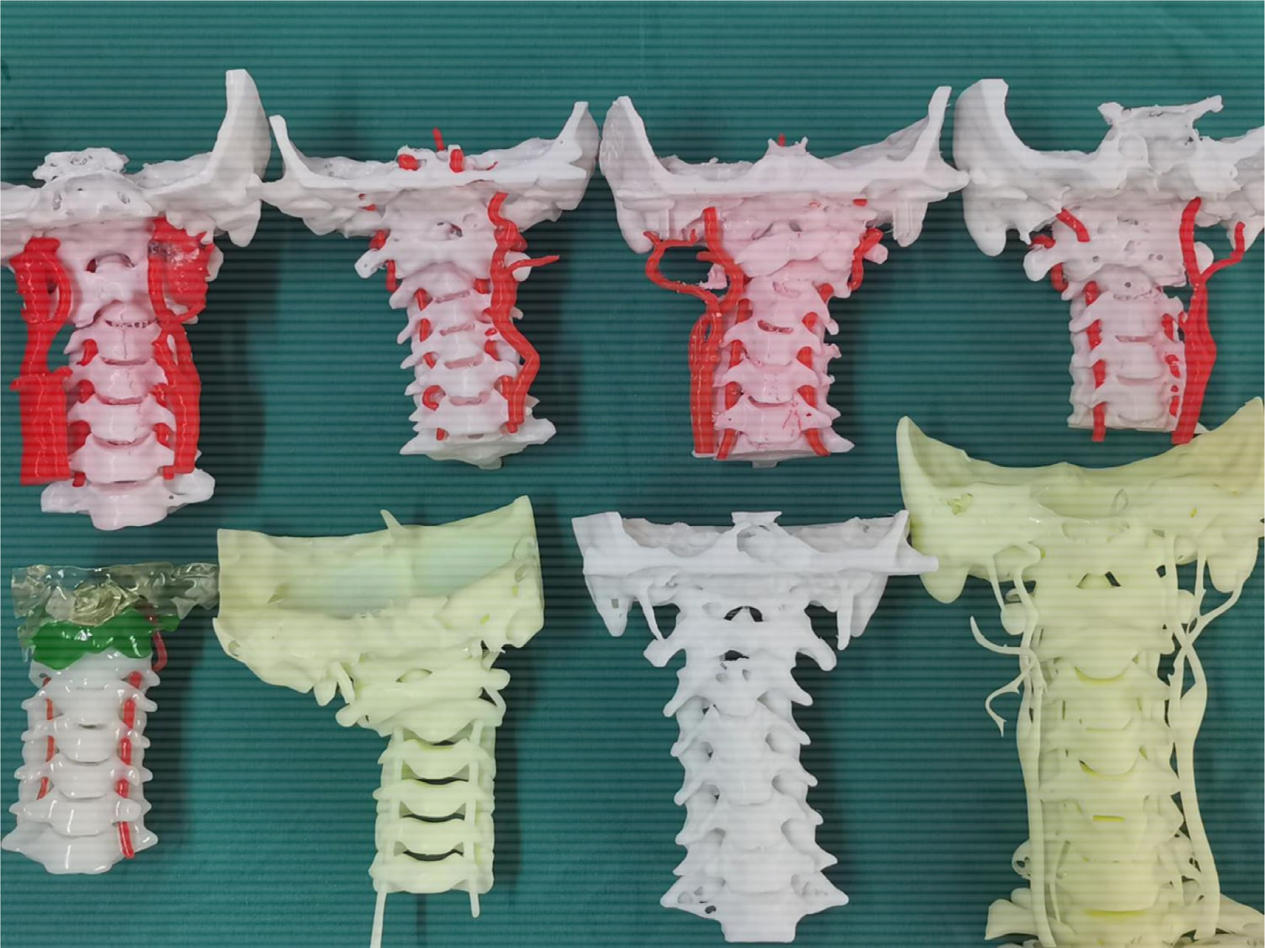
Different kinds of three‐dimensional models.

**Fig. 5:**
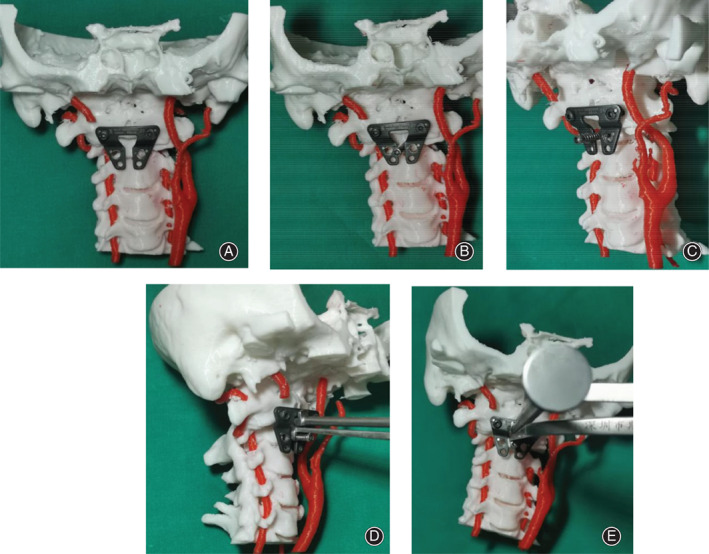
Key surgical procedures were simulated with the three‐dimensional (3D) model preoperatively (the right artery had been removed for surgery simulation). First, a transoral atlantoaxial reduction plate (TARP) and two screws were placed on C_1_ (A). Next, a provisional reduction screw was placed in the vertebrae of axis. (B, C). The most important part was the TARP specialized retractor, installed between a reduction screw and the complex comprised of the atlas and the plate, which was indispensable for the atlantoaxial reduction (D, E).

For the non‐3D group, surgeons could obtain information on the atlantoaxial anatomy abnormalities and undertake surgical planning based on the imaging data and previous experience. For the 3D group, the data of the above 3D digital models was saved in “ STL” format, which would be used to create a real‐size model using a 3D printer. (Figs 3 and 4) Surgeons could perform an *in vitro* operation simulation repeatedly with the 3D‐printed life‐size model. (Fig. 5) The 3D‐printed model brought to light the morphologic features of the IAAD from multiple angles and directions, so that surgeons were able to perform atlantoaxial reduction under direct visualization. It provided a helpful tool to assess the anatomy of the area in a 3D perspective and could be moved in different surgical positions to practice the entire surgical plan. Analyses of the type of IAAD, atlantoaxial joint abnormalities, the diameter and orientation of the pedicle, and the course of the vertebral artery could broaden our understanding of the TARP surgery. Visualization of the relationship between the atlas and the axis in a 3D perspective improved surgeons understanding of the IAAD. This information could assist in assessing the optimal site for screw implantation, which can help avoid injury to the vertebral artery. The 3D‐printed 1:1 scale model made it possible to preoperatively determine the sizes of the implants to ensure an optimal match between the bone surface and implants and to identify the region that needed to be drilled. For ease of use in the real operation, not only the location and size of the implant but also the length and orientation of the screw were assessed and recorded in the course of the simulation. Once the steps mentioned above were completed, the 3D‐printed individualized model was cleaned and sterilized for use during the surgery. (Fig. 3).

### Surgical Technique and Procedures

**Fig. 6:**
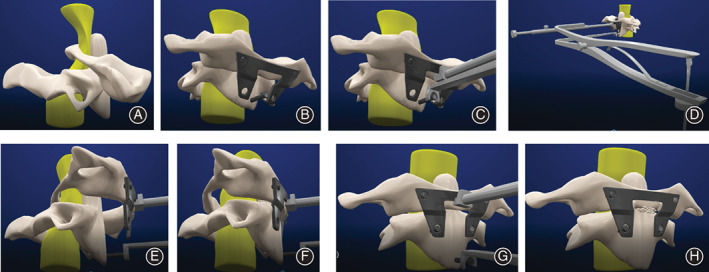
Technique and procedures for the transoral atlantoaxial reduction plate (TARP) surgery. Simulation of atlantoaxial dislocation is shown (A). During the surgery, a TARP plate and two screws were, respectively, installed on C1, then a provisional reduction screw was placed in the vertebrae of axis (B). Next, the TARP specialized retractor was installed between a provisional reduction screw and the complex comprised of the atlas and the plate (C). The atlas was ascending longitudinally and was pushed back by turning the nut on the top of the retractor (D, E). After the reduction of the atlas, two bicortical screws were placed at C_2_ and the reduction screw was pulled out (F, G). Finally, autogenous graft was implanted into the joint cavity (H).

All the surgery was performed by the same group. The preoperative preparation and surgical procedure were outlined in our previously published articles^5^, ^8^. Herein, we briefly describe the procedure of TARP surgery (Fig. 6).

**Step 1**. Anesthesia and position. Under general anesthesia with transnasal endotracheal intubation, the patient underwent skull traction with a weight of 4 to 10 kg in a supine position. Antibiotics were given intravenously as a preventive intervention in all patients half an hour before the operation.

**Step 2.** Approach and exposure. A midline incision of 4–5 cm was made along the posterior pharyngeal wall, and soft tissue was dissected to fully expose the surgical field, including bilaterally atlantoaxial joints and prevertebral structure.

**Step 3.** Release and resection. Tissue between the odontoid and the atlas were removed, such as scar tissue, contractural articular capsule, and hyperplastic osteotylus. After sufficient release, signs of atlantoaxial joint loosening and reduction could be seen gradually due to the sustained effect of skull traction.

**Step 4.** Fixation. A preselected TARP plate and two screws (Wego, China) were, respectively, installed on C1 and in the bilateral lateral masses of C1 to fix the plate (Fig. 6B), according to the preoperative surgical simulation and the anatomical structure dissected in the actual surgery. A provisional reduction screw was placed in the vertebrae of axis (Fig. 6B) and could move in the runner of the plate.

**Step 5.** Reconstruction. The TARP specialized retractor was installed between a provisional screw and the complex comprised of the atlas and the plate (Fig. 6C). The atlas was ascending longitudinally and was pushed back by turning the nut on the top of the retractor. Next, ideal reduction of the atlantoaxial joint was confirmed by intraoperative fluoroscopy. The other two or four preselected bicortical screws were placed at C_2_ to fix the TARP plate (Fig. 6G). Then, the provisional screw was pulled out. Finally, the atlantoaxial joint was distracted, the articular cartilage was denuded, an autogenous graft was implanted into the joint cavity through the plate window (Fig. 6H). Standard wound closure was performed, and the TARP plate was covered with muscular and mucosal layers of the posterior pharyngeal wall.

### Postoperative Management

A transnasal endotracheal catheter was used for 2 days after the operation, and a nasogastric tube was used for 1 week after the operation. Antibiotics were given intravenously as a preventive intervention in all patients for 5–7 days after surgery, and a neck collar was used for 3 months.

### Clinical and Radiographic Assessment

Preoperative and last follow‐up examinations, including X‐rays, CT, and MRI, were undertaken for radiographic assessments. Bony union and instrument failure were noted at every follow up. Parameters of clinic evaluation were noted as well, including JOA score, VAS score of neck pain, operation time, blood loss, times of fluoroscopy, and perioperative complications.

#### Atlas−Dens Interval

The atlas–dens interval (ADI) was used to determine whether there was atlantoaxial dislocation. It pointed to the distance from the posterior edge of the anterior tubercle of the atlas to the anterior edge of the odontoid process in lateral cervical radiographs in neutral position. An individual with an ADI over 3 mm is considered have an atlantoaxial dislocation.

#### Cervicomedullary Angle

The cervicomedullary angle (CMA) was used to measure the compression degree of the cervical spinal cord and medulla to estimate the decompression effect after surgery. The mid‐sagittal view of T2WI was selected as the measurement plane, with the junction of the medulla oblongata and the ventral side of the cervical spinal cord as the vertex. Parallel lines between the medulla oblongata and the ventral edge of the upper cervical spinal cord were drawn and the angle was measured. The normal range is 141.35°–166.99°. Less than 135°is considered spinal cord compression.

#### Clivus‐Canal Angle

The clivus‐canal angle (CCA) was used to measure the compression degree of the cervical spinal cord and medulla and to estimate the decompression effect after surgery. Measured on lateral cervical radiographs in neutral position, the CCA is the forward angle formed by the Wackenheim clivus baseline to the axial vertebral body and the posterior edge of the odontoid. The normal range of is 144.15°–169.47°. Less than 140° is considered spinal cord compression.

#### Japanese Orthopaedic Association

The Japanese Orthopaedic Association (JOA) score was used to evaluate the disability status. The JOA score system includes three sections: motion (eight points), sensory (six points), and bladder function (three points). Each score is added up to obtain the total score. Improvement rate = [(postoperation score – preoperation score)/(17 − preoperation score)] × 100%. Patients with a score of 100% are considered cured; 60%–99% means that patients have significantly improved; 25%–59% means that the treatment has been effective; less than 25% means that the treatment has been useless.

#### Visual Analogue Score

The visual analog score (VAS) was used to evaluate neck pain. A score of 0 means no pain, and a score of 10 means the most severe pain, which is unbearable.

### Statistical Analysis

All data were analyzed using SPSS 21.0 (IBM SPSS, USA). Ordinal variables were presented as medians and continuous variables as means and standard deviations. All the variables were compared between the two groups using the paired *t*‐test. Difference was considered statistically significant when *P* < 0.05.

## Results

### Demographic and Clinical Characteristics

**Fig. 7:**
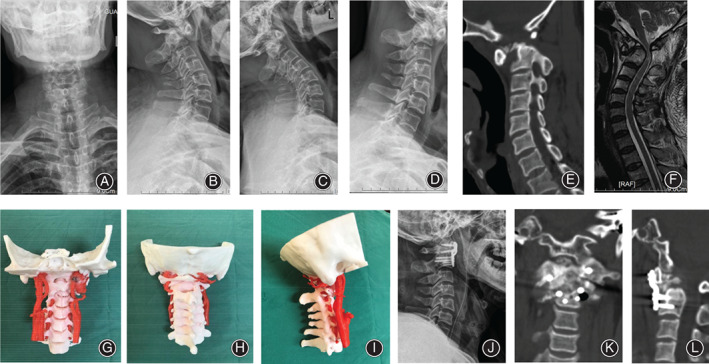
A 59‐year‐old male patient with irreducible atlantoaxial dislocation (IAAD) underwent transoral atlantoaxial reduction plate (TARP) surgery with the use of a three‐dimensional (3D) model. Preoperative images (A–F) show that the patient had atlantoaxial dislocation with compression of the spinal cord. The 3D model was made before the surgery, (G–I). Postoperative X‐ray and CT show atlantoaxial reduction after surgery (J–L).

**Fig. 8:**
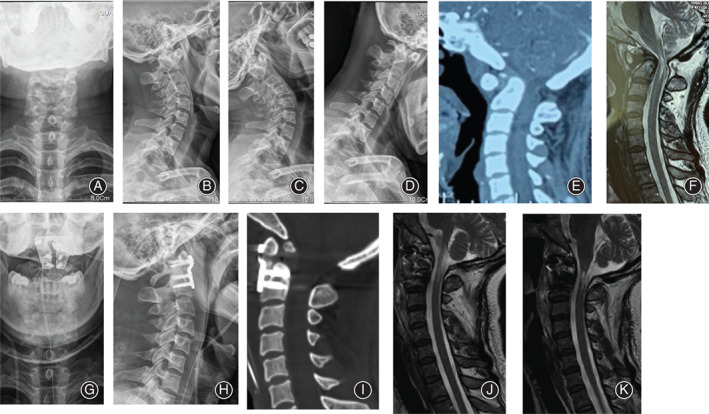
A 50‐year‐old female patient with irreducible atlantoaxial dislocation (IAAD) underwent transoral atlantoaxial reduction plate (TARP) surgery without the use of a three‐dimensional (3D) model. Preoperative images (A–F) show that the patient had an atlantoaxial dislocation with compression of the spinal cord. Postoperative images show atlantoaxial reduction and decompression of the spinal cord (G–K).

Transoral atlantoaxial reduction plate surgeries were successfully performed in 23 patients, whose symptoms were improved to different degrees (Fig. 7 and 8). Patient demographic and clinical characteristics for both groups are summarized in Table 1. There was no significant difference in the variables. The average follow‐up time was 16.08 months (12–25 months) in the 3D group and 16.45 months (12–26 months) in the non‐3D group. Patients with IAAD often had complex abnormalities of bone structure and an abnormal course of the vertebral artery in the craniovertebral junction (Table 2). In this study, an omnidirectional display of the atlantoaxial joint morphology was shown successfully through a 3D‐printed 1:1 scale model. In addition, the pre‐selected TARP plate of the model‐based preoperative simulation was the same as it used in the actual surgery. The surgery duration was 3.29 ± 0.45 h in the 3D group and 4.68 ± 0.90 h in the non‐3D group, and the estimated intraoperative blood loss was 131.67 ± 43.03 mL in the 3D group and 185.45 ± 42.28 mL in the non‐3D group. No patients received a blood transfusion. The intraoperative fluoroscopy time was 5.67 ± 0.89 in the 3D group and 7.91 ± 1.45 in the non‐3D group. Between the two groups, there was statistically significant difference for the three variables mentioned above.

**TABLE 1:** Demographic and clinical characteristics

Characteristics	3D group	Non‐3D group	Statistical value	*P*
Number of patients	12	11	/	/
Gender			/	/
Male	7	6		
Female	5	5		
Age (years), mean ± SD	58.17 ± 5.27	57.45 ± 6.36	0.304	0.772
History of trauma	10	9	/	/
Disease course (months)	16.50 ± 7.51	17.18 ± 5.17	0.249	0.804
Follow‐up time (months)	16.08 ± 4.50	16.45 ± 4.41	0.201	0.844

"/" means no needs to statistical comparison. 3D, three‐dimensional.

**TABLE 2:** Clinical and radiological characteristics of 23 patients with IAAD

No	Age	Sex	Other abnormalities	High‐riding VA	Dominance of VA	height of posterior atlas arch (>3.5 mm)	Time for preop skull Traction (d)	3D‐printed model
1	58	M	BI, partial C_1_ assimilation	Yes	Left	No	12	Yes
2	51	M	C_1_ assimilation, KFS	No	None	No	7	Yes
3	49	M	C_1_ assimilation, CM, KFS, small pedicle of C_2_	No	None	Yes	14	Yes
4	67	M	BI, OS, occipital dysplasia	No	Right	Yes	7	Yes
5	65	M	BI, defects of posterior arch of C_1_	No	Right	No	10	Yes
6	61	M	BI, OS	Yes	None	No	8	Yes
7	58	F	BI, small pedicle of C_2_	Yes	Right	No	9	Yes
8	55	F	C_1_ assimilation	No	Left	Yes	12	Yes
9	56	F	KFS	Yes	Right	No	7	Yes
10	56	F	C1 assimilation	No	Left	Yes	8	Yes
11	60	F	BI, C_1_ assimilation	Yes	Left	Yes	7	Yes
12	62	M	OS	NO	Right	Yes	10	Yes
13	68	M	C_1_ assimilation	Yes	Left	No	7	No
14	48	M	BI, small pedicle of C_2_	No	None	Yes	11	No
15	60	M	OS, partial C_1_ assimilation	Yes	None	No	15	No
16	49	M	OS, C_1_ assimilation	No	Right	No	13	No
17	51	M	KFS, small pedicle of C_2_	Yes	Right	Yes	8	No
18	54	M	BI, defects of posterior arch of C_1_	No	Left	Yes	9	No
19	57	F	OS, C_1_ assimilation	Yes	None	No	11	No
20	60	F	C_1_ assimilation	Yes	Right	Yes	10	No
21	59	F	BI, partial C_1_ assimilation	No	Left	No	8	No
22	62	F	BI, KFS	No	Left	Yes	7	No
23	64	F	OS, defects of posterior arch of C_1_	Yes	Left	No	7	No

BI, Basilar invagination; CM, Chiari malformation; IAAD, irreducible atlantoaxial dislocation; KFS, Klippel–Feil syndrome; OS, Os odontoideum; VA, vertebral artery; 3D, three‐dimensional.

### Clinical and Radiographic Outcomes

The clinical and radiographic outcomes are listed in Table 3. Preoperatively and at last follow up, the JOA score was 12.75 ± 1.96 and 15.92 ± 1.00 in the 3D group and 12.82 ± 2.04 and 15.91 ± 0.94 in the non‐3D group, and the VAS score was 3.08 ± 1.17 and 1.25 ± 0.87 in the 3D group and 3.00 ± 1.18 and 1.27 ± 0.97 in the non‐3D group, respectively. Radiographs were assessed. Preoperatively and at last follow up, the ADI was 7.75 ± 1.42 mm and 2.17 ± 0.72 mm in the 3D group and 7.64 ± 1.57 and 2.18 ± 0.75 in the non‐3D group, the CCA was 102.58° ± 6.10° and 140.83° ± 6.37° in the 3D group and 103.65° ± 7.87° and 139.31° ± 5.23° in non‐3D group, and the CMA was 115.17° ± 7.15° and 152.57° ± 6.80° in the 3D group and 116.60° ± 5.47° and 152.57° ± 4.81° in the non‐3D group, respectively. Preoperatively and at last follow up, JOA and VAS scores and ADI, CCA, and CMA were improved significantly within the two groups; however, no statistically difference was observed between the two groups. The two groups had similar clinical effectiveness.

**TABLE 3:** Clinical outcomes and radiological assessment

Characteristics	3D group	Non‐3D group	Statistic value	*P*
JOA score				
Preoperative	12.75 ± 1.96	12.82 ± 2.04	0.083	0.936
Postoperative	15.92 ± 1.00*	15.91 ± 0.94*	0.019	0.985
VAS score (neck pain)				
Preoperative	3.08 ± 1.17	3.00 ± 1.18	0.164	0.867
Postoperative	1.25 ± 0.87*	1.27 ± 0.97*	0.048	0.952
Surgery duration (h)	3.29 ± 0.45	4.68 ± 0.90	4.752	0.000
Blood loss (mL)	131.67 ± 43.03	185.45 ± 42.28	3.009	0.007
Fluoroscopy times (*n*)	5.67 ± 0.89	7.91 ± 1.45	4.511	0.000
Time of bone fusion (months)	7.25 ± 1.14	7.45 ± 1.04	0.442	0.658
ADI (mm)				
Preoperative	7.75 ± 1.42	7.64 ± 1.57	0.182	0.857
Postoperative	2.17 ± 0.72*	2.18 ± 0.75*	0.299	0.961
CMA (°)				
Preoperative	115.17 ± 7.15	116.60 ± 5.47	0.531	0.596
Postoperative	152.57 ± 6.80*	151.57 ± 4.81*	0.401	0.691
CCA (°)				
Preoperative	102.58 ± 6.10	103.65 ± 7.87	0.371	0.719
Postoperative	140.83 ± 6.37*	139.31 ± 5.23*	0.621	0.539

ADI, atlas–dens interval; CCA, clivus‐canal angle; CMA, cervicomedullary angle; JOA, Japanese Orthopaedic Association; Post, postoperative; Pre, preoperative; VAS, visual analogue score; 3D, three‐dimensional

* Compared to preoperatively, *P* < 0.05.

### Complications

The reduction of dislocation and bony fusion was satisfactory in both groups, and loss of reduction and loosening of instruments were not observed. However, some complications are inevitable. In the 3D group, 1 patient had a surgical site infection. On day 5 after the operation, the patient experienced a fever with a temperature fluctuation of 37.0–39.6°C; their extremity muscle strength was grade IV. An emergency MRI examination showed that an abscess was present on the ventral side of the spinal cord in the surgical site. An emergency revision operation was performed to remove the TARP device, and posterior occipitocervical fixation was conducted. The symptoms were relieved after intravenous injections of imipenem for 2 weeks after the revised operation. In the traditional group, 2 patients experienced mistakes in the placement of screws. Postoperative CT showed that the lengths of C1 screws were too long and the path of screws were not ideal, resulting that the screws had entered into the spinal canal. Fortunately, neither patient developed neurological symptoms related to the misplacement.

## Discussion

### The Importance of Three‐Dimensional‐Printed Model in Transoral Atlantoaxial Reduction Plate Surgery

Surgical management of IAAD, which is frequently accompanied by high surgical risk, remains a significant challenge^11^. For IAAD, one‐stage TARP surgery is an appropriate option to perform a direct decompression and fixation via the anterior approach, and is proven to be safe and effective^12^, ^13^. Precise preoperative understanding of patient information, including the inclination of atlantoaxial joints, the course of the vertebral artery, and the relationship between the location of the vertebral artery and the bone structure, is critical to the success of surgery. Currently, it is customary for surgeons to assess IAAD morphology preoperatively using 2D CT and 3D reconstruction imaging. Even though advanced imaging technology can be used to construct realistic 3D digital models, flat 3D images are still 2D images created using imaging technology. The added value of 3D digital models is limited, and diagnostic accuracy is not significantly improved. 3D models manufactured using 3D printing technology overcome this limitation and have significant potential in diagnosis and therapy. Surgeons will be able to better understand IAAD morphology with a 3D real‐size printed model. It has rapidly gained widespread popularity because of its potential to improve patient outcomes^14^, ^15^.

### Application of Three‐Dimensional‐Printed Model in Transoral Atlantoaxial Reduction Plate Surgery

**Fig. 9:**
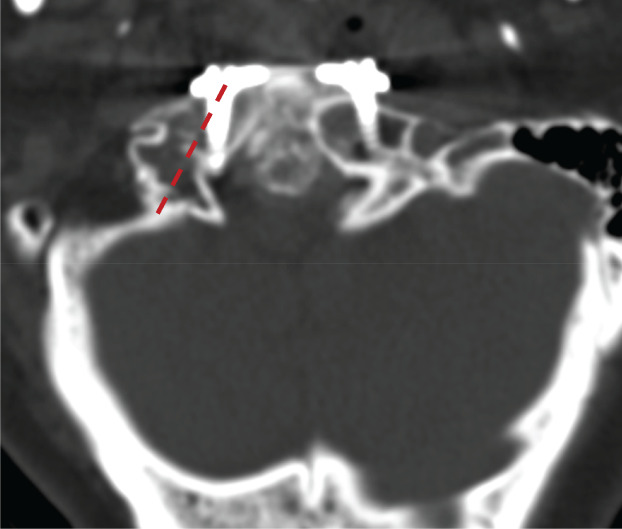
Misplacement of screws in the atlas. Postoperative CT scan shows that part of the screw (the right side) was inserted into the spinal canal. The red line shows the ideal screw path.

Preoperative information, such as osseous abnormalities, size and direction of the pedicle, location of the transverse foramen, and course of vertebral artery, is invaluable to spine surgeons who perform TARP surgery on patients with IAAD^16^. A 3D‐printed scale 1:1 model could be handled and moved in various positions, which provides essential information about the anatomy of the predefined region for surgery. This information helps surgeons to assess the optimal screw insertion site and avoid injury to the vertebral artery, which replaces the information obtained from 3D digital models and may also be better than virtual simulation. Surgery planning and simulation was conducted with the assistance of the 3D‐printed real‐size model, which was used as a template to choose the appropriate TARP plate, ensuring a perfect match between the bone surface and implants. Through repeat simulation, specific parameters for the plate (size and location) and screws (direction and the length) could be determined, eventually saving surgery time. With adequate preoperative surgery practice using a 3D‐printed model, surgical accuracy can be improved and potential complications related to surgery can be reduced. For instance, there were 2 patients in the traditional group who experienced misplacement of screws in the atlas, which mainly manifested as part of the screws being inserted into the spinal canal (Fig. 9). One patient in the 3D‐printed group had surgical site infection (SSI). Yin *et al*. specifically evaluated the rate of SSI after the transoral approach in 172 consecutive patients and found the incidence to be 3.5%^17^. However, the reasons are complicated and relate to age, gender, history of smoking, and history of diabetes^17^, ^18^. The SSI of the patient in the 3D‐printed group may be due to a history of smoking and malnutrition rather than the use of the model.

The two groups experienced similar clinical effectiveness. From our perspective, the same clinical outcomes demonstated that TARP surgery was an effective option for patients with IAAD. With the help of a 3D‐printed model, the risks of surgery can be effectively reduced (e.g. as a result of misplacement of screws or injury to vertebral artery). The 3D‐printed group was superior to the traditional group in preoperative surgery simulation. On the one hand, it is an educational tool for medical students. One the other hand, it is a valid means not only to promote surgeon–patient communication but also to improve patients' understanding of IAAD. The patient‐specific 3D‐printed model helps patients and family to understand the disease. Our study illustrated that the 3D‐printed model helped patients better understand the proposed surgical approach.

**Fig. 10:**
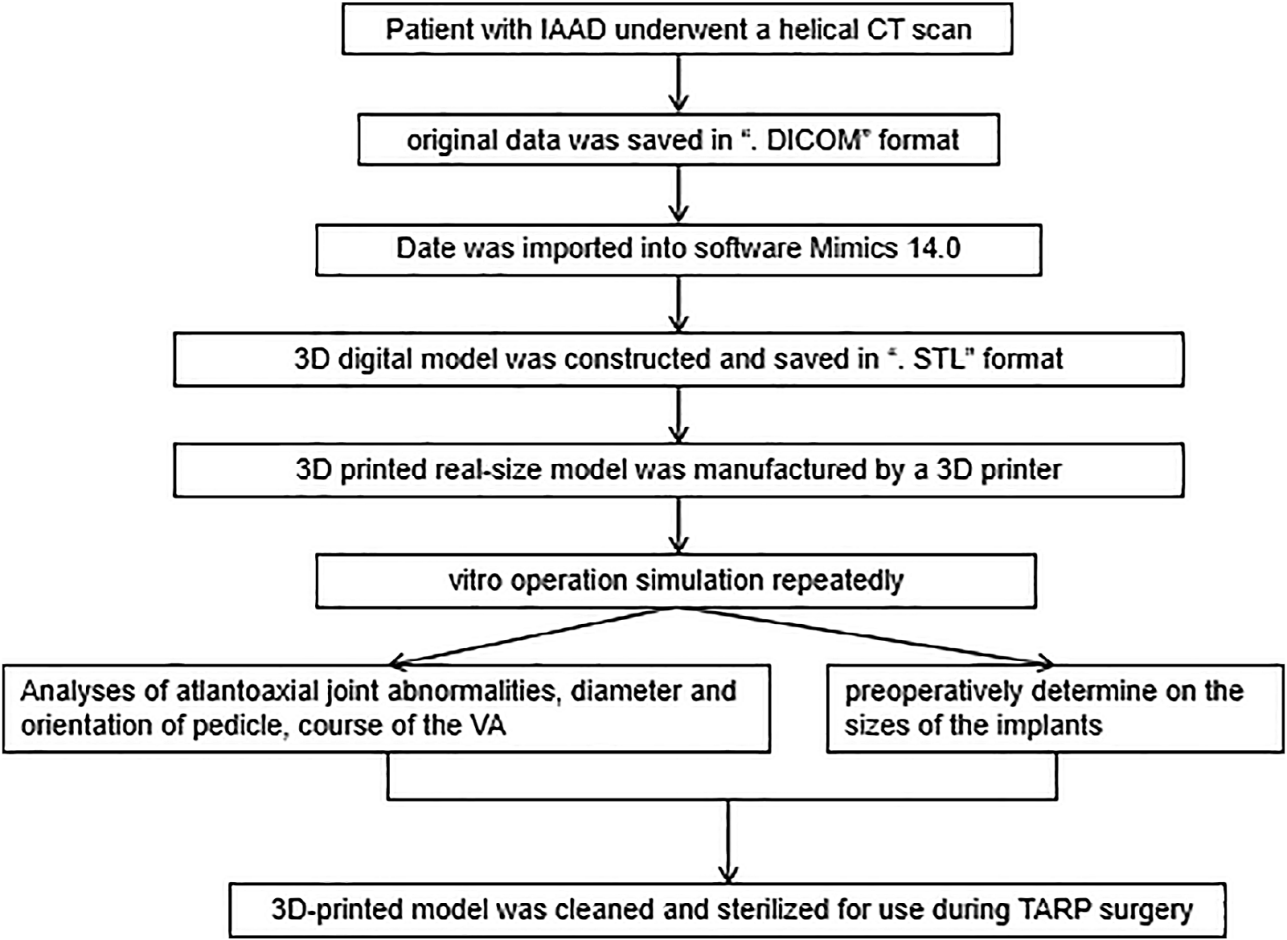
A working flowchart showed the steps to finish the preoperative plan in 3D group.

Further innovations are being developed to improve the materials used in 3D models. The 3D models have developed from single color to double color, even three colors or more, to improve the understanding of anatomical structures (Fig. 4). However, we cannot complete all the steps of the TARP surgery simulation, including the placement of bicortical screws at C2, because the joints of the models are immovable. Perhaps new kinds of models will rectify this. In the past, 3D printing technology was burdened by high costs and long production times, limiting its clinical application. Kim *et al*. reported that it took 2 to 3 days to obtain a solid model from data acquisition^19^. The printing procedure is gradually being simplified because of the rapid development of 3D printing technology and digital medicine. The length of time required for printing depends on the complexity of the patient. In our study, the production of a 3D‐printed model took, on average, 12 man‐hours after the CT scan, with an average cost of CNY1000. The time and expense of production were reduced in comparison to previous studies Fig. 10. This model is both cost‐effective and easy to construct and has become the basis for preoperative planning of IAAD in our institute.

In summary, the benefits of 3D‐printed real‐size models based on patients in this series are as follows. First, the 1:1 scale solid model provides a useful tool to assess the anatomy of the predefined region for operation, to help us obtain available information and develop a surgical strategy. Second, the 3D‐printed real‐size models are intuitive and convenient. They provide the ideal tool for surgeons to practice. They can be sterilized and used for comparison and reference during the surgical procedure. Third, the use of the models improves surgical accuracy and reduces operation duration and fluoroscopy times. Finally, the solid model is an effective tool to facilitate surgeon–patient communication and improve patients' understanding of IAAD.

### Limitations

This research had certain limitations. The most obvious limitation was the lack of a large sample. The advantages of 3D‐printed individualized guiding templates need to be further investigated in multicenter, large sample, controlled studies.

## Conclusion

Preoperative surgical simulation using a 3D‐printed real‐size model is a viable technique. The 3D‐printed model is an intuitive and effective aid for TARP surgery for treating IAAD. The 3D‐printed biomodel precisely replicates patient‐specific anatomy, and could potentially provide more useful information than 3D reconstructed images. It could be an invaluable aid for complicated craniovertebral junction surgeries.
